# Low rates of all-cause revision in displaced subcapital femoral neck fractures treated with hip hemiarthroplasty - a retrospective review of 4516 patients from a single institute

**DOI:** 10.1186/s12891-020-03725-0

**Published:** 2020-10-22

**Authors:** Chi-Yung Yeung, Shang-Wen Tsai, Po-Kuei Wu, Cheng-Fong Chen, Ming-Chau Chang, Wei-Ming Chen

**Affiliations:** 1 Department of Orthopaedics and Traumatology, Taipei Veterans General Hospital, No. 201, Sec 2, Shi-Pai Road, Taipei, 112 Taiwan, Republic of China; 2 Department of Orthopaedics, School of Medicine, National Yang-Ming University, No. 201, Sec. 2, Shipai Rd., Beitou District, Taipei City, 11217 Taiwan, Republic of China

**Keywords:** Femoral neck fractures, Austin Moore hemiarthroplasty, Bipolar hemiarthroplasty, Hemiarthroplasty failure, Conversion total hip replacement

## Abstract

**Background:**

Femoral neck fracture (FNF) is among the commonest fractures affecting the geriatric population. Hemiarthroplasty (HA) is a standard treatment procedure and has been performed by hip surgeons for decades. Recently, primary total hip replacement has proved advantageous for the treatment of such fractures.

The aim of this study is to retrospectively review all causes of failure of all patients who underwent HA in our institute and reevaluated whether HA remains a favourable choice of treatment for patients with displaced FNFs.

**Methods:**

A total of 4516 patients underwent HA at our centre from 1998 to 2017. The HA implants included unipolar and bipolar prostheses. Patients diagnosed with displaced FNF, underwent primary HA initially, required second revision procedures, and followed up for a minimum of 36 months were included in this study. Data were collected and comprehensively analysed.

**Results:**

In 4516 cases, 99 patients underwent second surgeries. The revision rate was 2.19%. Reasons for failure were acetabular wear (*n* = 30, 30.3%), femoral stem subsidence (*n* = 24, 24.2%), periprosthetic fracture (*n* = 22, 22.2%), infection (*n* = 16, 16.2%), and recurrent dislocation (*n* = 7, 7.1%). The mean follow-up period was 78.1 months. The interval between failed HA and revision surgery was 22.8 months.

**Conclusion:**

HA has a low revision rate and remains a favourable choice of treatment for patients with displaced FNFs.

**Levels of evidence:**

Level III, Retrospective Cohort Study, Therapeutic Study.

## Background

Femoral neck fracture (FNF) is among the commonest fractures affecting the geriatric population. In displaced fracture types, treatments include closed or open reduction and internal fixation, hemiarthroplasty (HA), and total hip replacement (THR). HA is a frequently recommended treatment and has been performed for decades [1]. Nevertheless, the use of primary THR has increased substantially in clinical research. Several randomised control trials have also demonstrated that for displaced FNF, THR results in superior functional outcomes to those of HA. However, THR is more expensive and results in higher complication rates. The clinical results appear contradictory [2–6]. The aim of this study is to retrospectively review all causes of failure of all patients who underwent HA in our institute. Whether HA remains a favourable choice of treatment for patients with displaced FNF can thereby be evaluated.

## Methods

**Fig. 1:**
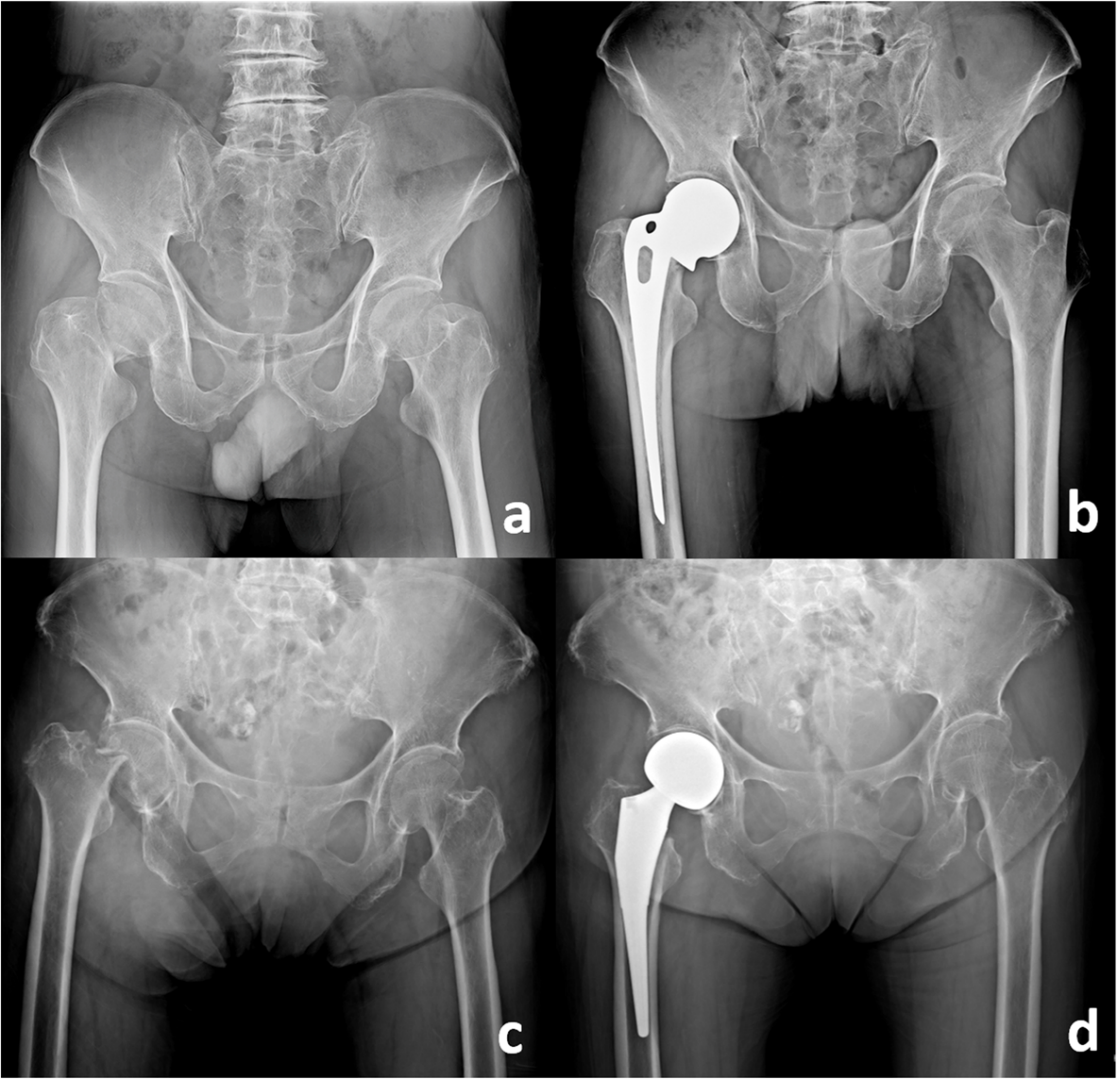
Displaced Femoral Neck Fractures and Hemiarthroplasties. **a** Patient diagnosed with right displaced femoral neck fracture; **b** Cemented Austin-Moore hemiarthroplasty; **c** Patient diagnosed with right displaced femoral neck fracture; **d** Cementless bipolar hemiarthroplasty

This was a retrospective cohort study and was performed at a single trauma centre. From 1998 to 2017, 4516 patients underwent hemiarthroplasty in our institute following a diagnosis of displaced FNF. The HA implants included the Austin Moore (unipolar monoblock) prosthesis and various bipolar systems (including the Zimmer, Osteonics, and United systems) (Fig. 1). Patients diagnosed with displaced FNF, underwent primary HA and second revision surgery, and followed up for at least 36 months were included in this study. Patients with multiple fractures, open fractures, pathological fractures, or paediatric fractures; patients who had received previous ipsilateral hip surgeries; and patients whose follow-up periods were insufficiently long were excluded. Data were collected in our database system and comprehensively analysed. The study was approved for publication by the institutional review board of our hospital.

Surgical procedures were performed by various surgeons according to the protocol of our department. Prophylactic antibiotics, including first-generation cephalosporin, were administered 30 min before skin incision and macrolides to patients with a penicillin allergy. Under spinal or general anaesthesia, patients were operated on in a lateral position, using either the anterolateral (Watson–Jones) approach or the posterior (Moore or Southern) approach. The prosthesis system was chosen according to the preference of the surgeon, and the use of cement fixation depended on bone quality and was decided intraoperatively. A portable radiograph of the hip joint was examined before the patient was transferred back to the ward unit. Oral analgesic agent and intravenous morphine (PRN) were administered for pain control if not contraindicated. Intravenous antibiotics were continuously administered every 8 h after surgery for 1 day and prolonged depending on the patient’s clinical condition.

Each patient had his or her own chart with detailed records, including personal data, the mechanism of injury and associated conditions, fracture type and classification, course of management, implantation details, fixation technique, surgical approach, and functional recovery process. Regular follow-ups were arranged after discharge for all patients. The anteroposterior and lateral radiograph views of the wound condition were evaluated during each outpatient department visit.

Statistical analysis was performed using SPSS version 24.0 statistical software (IBM-SPSS, Inc., Chicago, IL, USA). An independent *t* test, chi-square test, and multinomial logistic regression analysis were used. *P* < .05 was used to indicate statistical significance (*P < .05, **P < .01, ***P < .001).

## Results

Of 4516 patients, 99 were found to receive second revision surgery, including 5 open reductions and internal fixations, 18 revision hemiarthroplasties, and 76 conversion THRs. The revision rate of failed HA and the conversion rate of THR were 2.19% (99 of 4516) and 1.68% (76 of 4516), respectively. The average age of the patients at the time of the injury was 76.4 ± 8.7 years (range: 44–93 years). Of the patients, 57 were male and 42 were female. The physical health and associated medical conditions of the patients were rated based on the American Society of Anaesthesiologists’ (ASA) physical status classification: 32 patients were in class II, 41 patients were in class III, and 26 patients were in class IV. The mean body weight index (BMI) was 22.8 kg/m^2^ (range: 16.9–30.9). Of the fractures, 54 were left sided and 45 were right sided. The interval between injury and surgery was 1.8 ± 1.6 days (range: 1–8). The mean follow-up period was 78.1 ± 55.8 months (range: 40–219). Fourteen patients expired during the follow-up period due to infections (intra-abdominal infection and pneumonia were identified), malignancies, or cardiovascular diseases. All patients’ demographic data are summarised in Table 1.

**Table 1:** Patient demographics and clinical conditions. (Total *N* = 99)

**Age (year)**	
Mean + SD	76.4 ± 8.7
Range	44–93
**Gender**	
Male	57 (57.6)
Female	42 (42.4)
**BMI Index (kg/m2)**	
Mean + SD	22.8 ± 3.7
Range	16.9–30.9
**ASA Classification**	
Class 2	32 (32.3)
Class 3	41 (41.4)
Class 4	26 (26.3)
**Fracture Side**	
Left	54 (54.5)
Right	45 (45.5)
**Interval between ER Consultation to Surgery (days)**	
Mean + SD	1.8 ± 1.6
Range	1–8
**Duration of Follow-up (months)**	
Mean + SD	78.1 ± 55.8
Range	40–219
**Length of Hospital Stay (days)**	
Mean + SD	8.2 ± 7.0
Range	3–49
**Patients Expired during Follow-up Period**	
Number of Patients Expired	14 (14.1)
Duration between Primary HA to Expiration (months)	
Mean + SD	81.9 ± 43.0
Range	27–183
**Reasons of Expiration**	
Infection^a^	4 (28.6)
Malignancy	9 (64.3)
Cardiovascular disease	1 (7.1)

Data presented as N (%) unless otherwise stated in the table

^a^Infections were identified as intra-abdominal infection and pneumonia

**Fig. 2:**
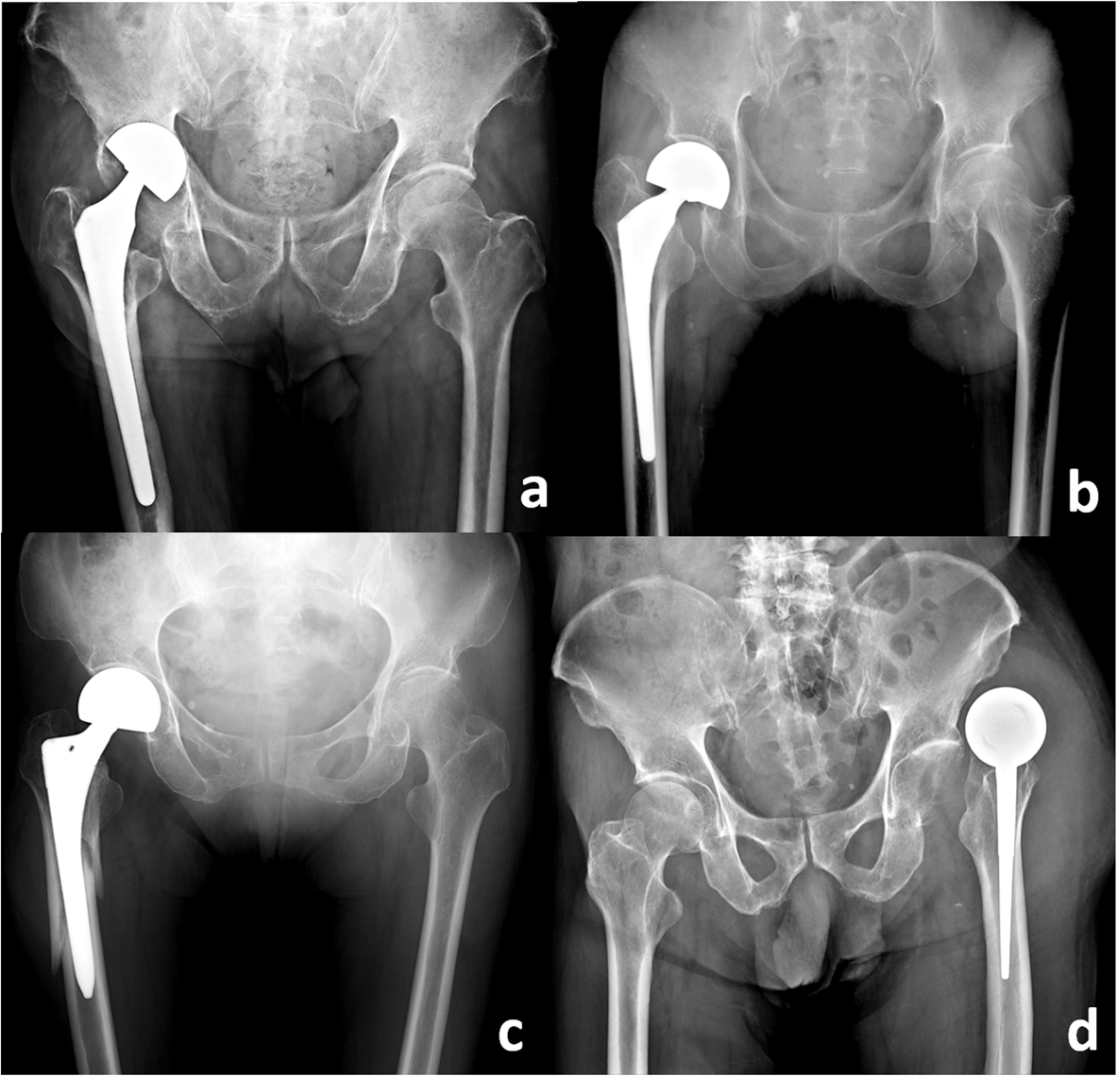
Reasons of failed hemiarthroplasty. **a** Acetabulum wearing with central migration; **b** Femoral stem subsidence; **c** Periprosthetic fracture; **d** Recurrent dislocation

The revision cases involved 24 and 75 patients who had undergone unipolar and bipolar HA, respectively. Of the HA procedures, 72 were cementless and 27 were cemented; 64 patients were operated on with the anterolateral (Watson–Jones) approach and 35 underwent the posterior (Moore or Southern) approach. The mean femoral cup size was 47.3 ± 3.6 mm (range: 40–54). Reasons for failure included acetabular wear (*n* = 30, 30.3%), femoral stem subsidence (*n* = 24, 24.2%), periprosthetic fracture (*n* = 22, 22.2%), infection (*n* = 16, 16.2%), and recurrent dislocation (*n* = 7, 7.1%) (Fig. 2). The interval between primary HA and revision surgery was 22.8 ± 30.0 months. Data are summarised in Tables 2, 3 and 4.

**Table 2:** Surgical details, reasons of failed hemiarthroplasty and types of revision surgery

**Implant of Hemiarthroplasty**	
Unipolar (Austin-Moore) Hemiarthroplasty	24 (24.2)
Bipolar Hemiarthroplasty	75 (75.8)
**Fixation Technique**	
Cementless	72 (72.7)
Cemented	27 (27.3)
**Surgical Approach**	
Anterolateral (Watson-Jones) Approach	64 (64.6)
Posterior (Moore/ Southern) Approach	35 (35.4)
**Femoral Cup Size (mm)**	
Mean + SD	47.3 ± 3.6
Range	40–54
**Reasons of Failed Hemiarthroplasty**	
Acetabulum Wearing	30 (30.3)
Femoral Stem Subsidence	24 (24.2)
Periprosthetic Fracture	22 (22.2)
Infection	16 (16.2)
Recurrent Dislocation	7 (7.1)
**Interval between Primary HA to Second Surgery (months)**^a^	
Mean + SD	22.8 ± 30.0
Range	1–176

Data presented as N (%) unless otherwise stated in the table

^a^Interval between Primary Surgery (Unipolar / Bipolar Hemiarthroplasty) to Second Surgery (Open Reduction and Internal Fixation / Revision Hemiarthroplasty / Conversion Total Hip Replacement

**Table 3:** Comparison between different reasons of failed hemiarthroplasties

Total *N* = 99	Acetabulum wearing	Femoral stem subsidence	Recurrent dislocation	Periprosthetic fracture	Infection	*p-value*
	*n* = 30	*n* = 24	*n* = 7	*n* = 22	*n* = 16	
**Age (year)**						
< 80	19 (63.3)	11 (45.8)	4 (57.1)	11 (50.0)	11 (68.8)	0.564
≥ 80	11 (36.7)	13 (54.2)	3 (42.9)	11 (50.0)	5 (31.3)	
**Gender**						
Male	17 (56.7)	14 (58.3)	2 (28.6)	13 (59.1)	11 (68.8)	0.552
Female	13 (43.3)	10 (41.7)	5 (71.4)	9 (40.9)	5 (31.3)	
**BMI (kg/m**^**2**^**)**						
≤ 18.5	4 (15.4)	3 (14.3)	0 (0)	2 (13.3)	4 (26.7)	0.298
> 18.5–25	13 (50.0)	10 (47.6)	4 (80.0)	13 (86.7)	7 (46.7)	
> 25–30	7 (26.9)	7 (33.3)	1 (20.0)	0 (0)	4 (26.7)	
> 30–40	2 (7.7)	1 (4.8)	0 (0)	0 (0)	0 (0)	
**ASA Classification**						
Type 2	12 (40.0)	8 (33.3)	3 (42.9)	4 (18.2)	5 (31.3)	0.425
Type 3	14 (46.7)	8 (33.3)	2 (28.6)	9 (40.9)	8 (50.0)	
Type 4	4 (13.3)	8 (33.3)	2 (28.6)	9 (40.9)	3 (18.8)	
**Implant of Hemiarthroplasty**						
Unipolar HA	8 (26.7)	5 (27.8)	2 (33.3)	0 (0)	3 (21.4)	0.987
Bipolar HA	22 (73.3)	13 (72.2)	4 (66.7)	8 (100)	11 (78.6)	
**Fixation Technique**						
Cementless	21 (70.0)	18 (75.0)	4 (57.1)	17 (77.2)	12 (75.0)	0.859
Cemented	9 (30.0)	6 (25.0)	3 (42.9)	5 (22.7)	4 (25.0)	
**Surgical Approach**						
Anterolateral (Watson-Jones)	20 (66.7)	11 (45.8)	6 (85.7)	12 (59.1)	14 (87.5)	0.060
Posterior (Moore/ Southern)	10 (33.3)	13 (54.2)	1 (14.3)	9 (40.9)	2 (12.5)	
**Femoral Cup Size (mm)**						
≤ 45	8 (26.7)	9 (37.5)	4 (57.1)	7 (35.0)	4 (25.0)	0.315
45–50	15 (50.0)	8 (33.3)	1 (14.3)	4 (20.0)	4 (25.0)	
≥ 50	7 (23.3)	7 (29.2)	2 (28.6)	9 (45.0)	8 (50.0)	
**Primary to Second Surgery (year)**						
≤ 0.5	4 (13.3)	7 (29.2)	5 (71.4)	14 (63.6)	4 (25.0)	0.001***
> 0.5–1	1 (3.3)	7 (29.2)	0 (0.0)	1 (4.5)	4 (25.0)	
> 1–2	8 (26.7)	5 (20.8)	1 (14.3)	1 (4.5)	4 (25.0)	
≥ 3	17 (56.7)	5 (20.8)	1 (14.3)	6 (27.3)	4 (25.0)	

Data presented as N (%) unless otherwise stated in the table

*p-value < 0.05, **p-value < 0.01, ***p-value < 0.001

**Table 4:** Comparison between unipolar (Austin Moore) hemiarthroplasty and bipolar hemiarthroplasty

Total *N* = 99	Unipolar	Bipolar	*p-value*
	*n* = 24	*n* = 75	
**Age (year)**			
< 80	0(0)	56(74.7)	0.001***
≥ 80	24(36.7)	19(25.3)	
**Gender**			
Male	20(83.3)	37(49.3)	0.003**
Female	4(16.7)	38(50.7)	
**BMI (kg/m**^**2**^**)**			
≤ 18.5	5(25.0)	8(12.9)	0.51
> 18.5–25	11(55.0)	36(58.1)	
> 25–30	3(15.0)	16(25.8)	
> 30–40	1(5.0)	2(3.2)	
**ASA Classification**			
Type 2	8(33.3)	24(32.0)	0.985
Type 3	10(41.7)	31(41.3)	
Type 4	6(25)	20(26.7)	
**Surgical Approach**			
Anterolateral (Watson-Jones)	16(66.7)	48(64.0)	0.812
Posterior (Moore/ Southern)	8(33.3)	27(36.0)	
**Cup Size (mm)**			
≤ 45	3(13.0)	29(39.2)	0.066
45–50	10(43.5)	22(29.7)	
≥ 50	10(43.5)	23(31.1)	
**Primary to Second Surgery (year)**			
0.5y	5(20.8)	29(38.7)	0.08
1y	1(4.2)	12(16.0)	
2y	7(29.2)	12(16.0)	
≥ 3y	11(45.8)	22(29.3)	
**Reasons of Failed Hemiarthroplasty**			
Acetabulum Wearing	8(33.3)	22(29.3)	0.986
Femoral Stem Subsidence	5(20.8)	19(25.3)	
Recurrent Dislocation	2(8.3)	5(6.7)	
Periprosthetic Fracture	5(20.8)	17(22.7)	
Infection	4(16.7)	12(16.0)	

Data presented as N (%) unless otherwise stated in the table

*p-value < 0.05, **p-value < 0.01, ***p-value < 0.001

## Discussion

The National Health Insurance Research Database of Taiwan documents more than 100,000 hip fracture diagnoses that have caused more than 2000 in-hospital mortalities every year. Along with the trend of rapid population aging, standard management for hip fractures is a prominent theme and represents a challenge for orthopaedic surgeons [7, 8].

For displaced FNFs, HA is the standard treatment. However, one study reported that the rate of THR use as a primary treatment option significantly increased from 0.7 to 7.7% between 1999 and 2011. Younger patients are being treated with THRs due to their superior mobility and range of joint motion [9, 10]. Clinical research has also shown that THR is superior to HA. For example, Ravi reported that THR is associated with lower revision surgery rates and significantly reduces the total costs of hospitalisation. Nevertheless, Sonaje et al. stated that HA yielded superior functional outcomes and cost-effectiveness to THR. Wang et al. also reported lower proportional hazard values for reoperation in patients treated with HA compared with those treated with a THR [2–6]. Although clinical results are controversial, the surgical procedure of HA has a much shorter duration, results in less tissue damage and exposure, reduces blood loss, improves primary stability, and reduces dislocation and complication rates compared with THR. Moreover, catastrophic metallosis and osteolysis are rarely observed in hemiarthroplasty. These advantages of HA ostensibly make it a superior treatment for older adults with various underlying comorbidities [1, 5].

Some concerns in relation to HA have been discussed in other studies: The reoperation rate for failed HA is reportedly as high as 24%, and the problem of acetabular wear has been noted as the primary cause of HA failure [11–15]. These concerns might provide additional motivation for the recommendation of primary THR for FNF displacement. However, in the present study, the HA failure rate and the THR conversion rate were 2.19 and 1.68%, respectively. In this study, the reasons for the failure of HA were acetabular wear (30.3%), femoral stem subsidence (24.2%), periprosthetic fracture (22.2%), infection (16.2%), and recurrent dislocation (7.1%). The prevalence of acetabular wear, femoral stem subsidence, and periprosthetic fracture were similar within the first 6 months after primary HA according to a multinomial logistic regression analysis. The main cause of early failure was periprosthetic fracture, but the cause of failure became evenly distributed for all 5 groups as time elapsed, and the rates of acetabular wear gradually increased in patients followed up for more than 3 years. A significant difference was demonstrated using a statistical analysis (*P* < .001***). The aggressive prevention of postoperative trauma is ostensibly more critical than is long-term acetabular wear.

No significant difference was noted in the comparison among the groups for the 5 HA failure types in terms of age, sex, BMI index, ASA classification, prosthesis use, fixation technique, surgical approach, and femoral cup size. The risk factor of HA failure was not identified. Peter et al. found that higher ASA scores and BMI indexes (> 40) are strong predictors of revision THR requirement, but similar results were not obtained in our data analysis. Further studies are required to determine the major predictors of HA failure [16].

The risk of periprosthetic fractures when using cemented or cementless stems are currently discussed. Olof GS et al. stated cementless femoral stems are not recommended for the treatment of FNFs in geriatrics high number of due to late-occurring periprosthetic fractures [17]. However, James K et al. reported periprosthetic fractures occur equally in cemented and cementless stems under the Vancouver classification [18]. The use of cemented or cementless stems for FNFs remains another controversial issue. From the multinomial logistic regression analysis of this study, the odds ratio of risk of periprosthetic fracture is 2.155 in the cementless group comparing to the cemented group (after adjustment of age and gender), but no significance difference (*P* = 0.282, CI = 0.532–8.736) is noted. The result of analysis is presented in Table 5. Further studies are needed for to evaluate the fixation technique of femoral stem in this geriatric population.

**Table 5:** Multivariable logistic regression analysis – cemented and cementless fixation for femoral stem in hip hemiarthroplasty

	*B* value	*p*-value	Multivariable-adjusted OR (95% CI)^a^
Age	0.054	0.173	1.055 (0.977–1.140)
Gender (Female ref.)	0.102	0.865	1.107 (0.342–3.579)
**Periprosthetic Fracture** (Acetabulum Wearing ref.)	0.768	0.282	**2.155** (0.532–8.736)

*OR* odds ratio, *CI* confidence interval

*p-value < 0.05, **p-value < 0.01, ***p-value < 0.001

^a^Data adjusted for age and gender

This study has limitations. First, it was a single-centre retrospective cohort study. Second, surgeries were performed by different surgeons and using different surgical approaches, fixation methods, and prosthesis systems. More comprehensive research and randomised control studies are required to elucidate these results.

## Conclusion

On the basis of the encouraging mid- to long-term outcomes in this population, we consider that hemiarthroplasty remains a favourable choice of treatment for patients with displaced FNFs.

## Data Availability

The datasets analysed during the current study are not publicly available due to the health policy of protection of patient privacy announced by the Ministry of Health and Welfare of Taiwan but are available from the corresponding author on reasonable request.

## Abbreviations

FNF: Femoral neck fracture
HA: Hemiarthroplasty
THR: Total hip replacement
ORIF: Open reduction and internal fixation
CRIF: Closed reduction and internal fixation
BMI: Body Mass Index
ASA: American Society of Anaesthesiologists
